# Protein Consumption and the Elderly: What Is the Optimal Level of Intake?

**DOI:** 10.3390/nu8060359

**Published:** 2016-06-08

**Authors:** Jamie I. Baum, Il-Young Kim, Robert R. Wolfe

**Affiliations:** 1 Department of Food Science, University of Arkansas, 2650 N. Young Ave, Fayetteville, AR 72704, USA; 2 Department of Geriatrics, the Center for Translational Research on Aging and Longevity, Donald W. Reynolds Institute on Aging, College of Medicine, The University of Arkansas for Medical Sciences, Little Rock, AR 72205, USA; iykim@uams.edu (I.-Y.K.); rwolfe2@uams.edu (R.R.W.)

**Keywords:** protein, aging, muscle, requirements, anabolic response, protein synthesis, elderly

## Abstract

Maintaining independence, quality of life, and health is crucial for elderly adults. One of the major threats to living independently is the loss of muscle mass, strength, and function that progressively occurs with aging, known as sarcopenia. Several studies have identified protein (especially the essential amino acids) as a key nutrient for muscle health in elderly adults. Elderly adults are less responsive to the anabolic stimulus of low doses of amino acid intake compared to younger individuals. However, this lack of responsiveness in elderly adults can be overcome with higher levels of protein (or essential amino acid) consumption. The requirement for a larger dose of protein to generate responses in elderly adults similar to the responses in younger adults provides the support for a beneficial effect of increased protein in older populations. The purpose of this review is to present the current evidence related to dietary protein intake and muscle health in elderly adults.

## 1. Introduction

The United States is experiencing considerable growth in its elderly adult population. By 2015, the population aged 65 and over is projected to reach nearly 84 million [1]. Maintaining independence, quality of life, and health is crucial for elderly adults [2]. One of the major threats to living independently is the loss of muscle mass, strength, and function that progressively occurs with aging, known as sarcopenia [2,3]. A loss or reduction in skeletal muscle function often leads to increased morbidity and mortality either directly, or indirectly, via the development of secondary diseases such as cardiovascular disease, diabetes, and obesity [3,4]. The prevalence of obesity among elderly adults has also increased over the last several decades. For example, the prevalence of obesity among men aged 65–74 increased from 31.6% in 1999–2002 to 41.5% in 2007–2010. Between 2007 and 2010, approximately 35% of adults aged 65 and over were obese [5]. One reason for the increase in obesity could be due to body composition shifts that occur as we age, resulting in a higher percentage of body fat and decreases in muscle mass with age [6]. Both sarcopenia and obesity act synergistically, which increases the risk of negative health outcomes and earlier onset of disability [2].

Nutrition plays an essential role in the health and function of elderly adults [7]. Inadequate nutrition can contribute to the development of both sarcopenia and obesity [3,8]. As life expectancy continues to rise, it is important to consider optimal nutritional recommendations that will improve health outcomes, quality of life, and physical independence in elderly adults [5]. Several studies have identified protein as a key nutrient for elderly adults (reviewed in [3,8]). Protein intake greater than the recommended amounts may improve muscle health, prevent sarcopenia [9], and help maintain energy balance, weight management [10], and cardiovascular function [11,12,13]. Benefits of increased protein intake include improved muscle function and the prevention onset of chronic diseases, which can increase quality of life in healthy elderly adults [3]. Therefore, the purpose of this review is to present the current evidence related to dietary protein intake and muscle health in elderly adults.

## 2. Optimal Protein Intake for Elderly Adults

### 2.1. Dietary Protein Recommendations

Traditionally, protein recommendations have been based on studies that estimate the minimum protein intake necessary to maintain nitrogen balance [3,8]. However, the problem with relying on these results is that they do not measure any physiological endpoints relevant to healthy aging, such as muscle function. The current dietary recommendations for protein intake include the dietary reference intakes (DRI) for macronutrients, which include an estimated average requirement (EAR), a recommended dietary allowance (RDA) and an acceptable macronutrient distribution range (AMDR) [14]. In the case of daily protein intake, the EAR for dietary protein is 0.66 g/kg/day and the Food and Nutrition Board recommends an RDA of 0.8 g/kg/day for all adults over 18 years of age, including elderly adults over the age of 65. The RDA for protein was based on all available studies that estimate the minimum protein intake necessary to avoid a progressive loss of lean body mass as determined by nitrogen balance [3,8]. The Food and Nutrition Board recognizes a distinction between the RDA and the level of protein intake needed for optimal health. Therefore, the recommendation for the ADMR includes a range of optimal protein intakes in the context of a complete diet (10%–35% of daily energy intake come from protein [14]), which makes the ADMR more relevant to normal dietary intake than the RDA [3].

### 2.2. Protein Requirements for Elderly Adults

Experts in the field of protein and aging recommend a protein intake between 1.2 and 2.0 g/kg/day or higher for elderly adults [3,8,15]. The RDA of 0.8 g/kg/day is well below these recommendations and reflects a value at the lowest end of the AMDR. It is estimated that 38% of adult men and 41% of adult women have dietary protein intakes below the RDA [16,17].

Most published results, based on data from either epidemiological or short-term studies, indicate a potential beneficial effect of increasing protein intake in elderly adults. These data demonstrate that elderly adults, compared with younger adults, are less responsive to low doses of amino acid intake [18]. However, this lack of responsiveness in healthy older adults can usually be overcome with higher levels of essential amino acid (EAA) consumption [18]. This is also reflected in studies comparing varying levels of protein consumption [19], suggesting that the lack of muscle responsiveness to lower doses of protein intake in elderly adults can be overcome with a higher level of protein intake. The requirement for a larger dose of protein to generate responses in elderly adults similar to the responses in younger adults provides the support for a beneficial effect of increased protein in older populations [8].

The mechanism by which dietary protein affects muscle is through the stimulation of muscle protein synthesis and/or suppression of protein breakdown by the absorbed amino acids consumed in the diet [20,21]. There appears to be an EAA threshold when it comes to stimulating muscle protein synthesis. Ingestion of relatively small amounts of EAA (2.5, 5 or 10 g) appears to increase myofibrillar protein synthesis in a dose-dependent manner [22]. However, a larger dose of EAA (20–40 g) fail to elicit an additional effect on protein synthesis in young and older subjects. Similar results were observed after the ingestion of either 113 or 340 g of lean beef containing 10 or 30 g EAA, respectively [23]. Despite a threefold increase in EAA content, there was no further increase in protein synthesis in either young or older subjects following consumption of 340 g *versus* 113 g of protein. There are fewer data regarding the response of protein breakdown to different levels of protein or amino acid intake. The balance between protein synthesis and breakdown is discussed in more detail below.

### 2.3. Essential Amino Acid Requirements for Aging Adults

Essential amino acids, especially the branched-chain amino acid leucine, are potent stimulators of muscle protein synthesis. Studies have focused on the stimulation of muscle protein synthesis via the protein kinase mTORC1 (mechanistic target of rapamycin complex 1) [24,25,26], but the *in vivo* significance of this mechanism as a regulator of the rate of protein synthesis in human subjects is not yet proven. Several studies demonstrate that maximal stimulation of muscle protein synthesis is possible with 15 g of EAA (reviewed in [20]). This translates to ~35 g of high quality protein per meal delivering ~15 g of EAA. A larger amount of lower quality protein, which contains a lower content of EAA, would be required to achieve the same functional benefits. The addition of nonessential amino acids to a supplement containing EAA does not result in additional stimulation of muscle protein synthesis [27], indicating that the quality of the protein, or its amino acid profile, is a key determinant of the functional potential of protein in muscle health. This is supported by several studies demonstrating that the ingestion of milk proteins, compared with the ingestion of soy protein stimulates muscle protein synthesis to a greater extent after resistance exercise, owing to the higher content of EAA in milk protein [28,29,30,31]. The data from the Health, Aging and Body Composition study support these findings [31], showing that intake of animal protein (with greater content of EAA), but not plant protein, was significantly associated with the preservation of lean body mass over three years in older adults [31]. In that study, individuals in the highest quintile of protein intake had 40% less loss in lean body mass than those in the lowest quintile of protein intake [31].

### 2.4. The Importance of Protein Quality

When considering protein intake, it is also important to consider total energy intake. Age is associated with a progressive decline in basal metabolic rate (BMR) at a rate of 1%–2% per decade after 20 years of age [32,33,34]. This reduction in BMR is closely associated with the loss in fat-free mass, including muscle, and the gain of less metabolically active fat [35] that occurs as we age [33]. In fact, studies suggest that BMR adjusted for the change in fat-free mass is 5% lower in elderly adults compared to younger adults [35]. This implies that aging adults require a lower daily energy intake. However, the extent to which BMR may increase or decrease with age depends on the balance between weight gain with age, tending to increase BMR, and aging, which decreases BMR [35].

Although older adults typically eat less than younger adults, including less protein [15,16], it is important for aging adults to consider total caloric intake when choosing a protein source to incorporate in the diet. The discrepancies in quality between animal and plant protein sources go beyond the amino acid profiles. When the energy content of the protein source is accounted for, the caloric intake needed to meet the EAA requirements from plant sources of protein is considerably higher than the caloric intake from animal sources of protein [36]. This is important to consider since obesity, especially with aging, is a major public health concern. Obesity is the most predominant factor limiting mobility in the elderly [37].

### 2.5. Dietary Protein and Muscle Anabolic Response in Elderly Adults

There is abundant evidence that muscle plays a central role in the prevention of many chronic diseases, including diabetes and obesity [38]. In addition, evidence that optimal health for elderly adults is dependent on maintaining muscle mass is emerging [3,8]. EAAs are the primary nutrients responsible for the maintenance of muscle mass and function, but elderly individuals have reduced anabolic sensitivity to amino acids (termed anabolic resistance). An increasing amount of evidence suggests that a minimum threshold of EAA needs to be reached to elicit an anabolic muscle response, and older individuals require a higher concentration of amino acids compared to younger individuals.

Optimal protein intake per meal can be defined as the minimal dose of protein intake that results in the maximal anabolic response and thus can help maintain or improve muscle mass (reflected as lean body mass) and function over time. It has been reported that the optimal dose of dietary protein consumption in a meal that results in a near maximal anabolic response is ~35 g/meal [23] or 0.40 g/kg/meal of high-quality protein in elderly adults [19], translatable to 1.2 g/kg/day or 96 g/day for an 80 kg elderly adults. The optimum amount for elderly adults (0.24 g/kg/meal) is approximately 70% greater than that for young adults (0.8 g/kg/day) [19], indicating an age-associated anabolic resistance to dietary protein. It is likely that elderly individuals need more protein intake to achieve a maximal anabolic response per meal considering the varying degrees of quality of protein eaten in the real world. In a typical American diet, the consumption of the majority of total daily protein intake skews toward dinner (~50% of total amount; ~40–60 g protein) [16,17,39] that clearly exceeds the “optimal” protein dose (*i.e.*, ~35 g protein/meal) without extra stimulation of anabolic response. This led to an interesting hypothesis that spreading daily protein intake evenly throughout the day can result in a greater cumulative anabolic response than the skewed pattern of protein intake [40]. If this is the case, elderly adults can gain benefits regarding improvement in muscle mass and strength, and related functions, simply by adopting even distribution pattern of equal amounts of protein intake [40]. However, the rationale behind this hypothesis is largely incorrect, as the hypothesis was solely based on data on muscle protein synthesis (MPS), which is only one half of the equation determining net anabolic response (*i.e.*, net anabolic response = protein synthesis minus protein breakdown).

The significance of simultaneous measurement of both protein synthesis and breakdown is dependent on a number of catabolic conditions (*i.e.*, loss of muscle mass over time) such as type I diabetes, cancer cachexia, and burn injury, in which the rate of protein synthesis is typically not blunted but actually normal or often increased [41], due largely to the increased availability of amino acids secondary to an accelerated rate of protein breakdown. This issue is important when quantifying the net anabolic response to dietary protein intake. Furthermore, although net anabolic response at the muscle level is the most relevant physiological response, the whole body is potentially involved in the anabolic response to protein ingestion, as approximately half of the total body protein turnover occurs at non-muscle tissues, particularly gut tissue [42]. Thus, determination at the muscle level could underestimate total anabolic response. For example, a large portion of the amino acids absorbed from a meal is retained in gut proteins that turn over rapidly [42,43], particularly following a mixed meal, due largely to a systemic insulin response [42]. Those amino acids can, in turn, be released into the blood over time as a result of a protein breakdown and be used for incorporation into new proteins in muscle. This is of particular importance in situations where older adults consume a protein intake greater than the amount that stimulates a maximal MPS.

Consistent with this notion, our recent findings showed that similar MPS responses were achieved by two doses of protein intake (40 g *vs.* 70 g), while a greater net protein synthesis at whole-body level was achieved with a meal containing 70 g of protein due to the suppression of breakdown amplifying the anabolic effect of the stimulation of synthesis [44]. Furthermore, we have directly tested the “distribution” hypothesis at two protein levels (0.8 g or 1.5 g protein/kg/day) in mixed meals and found no beneficial effects of an even distribution pattern of protein intake on net anabolic response at whole-body level and MPS [45]. Instead, we found the higher protein intake (*i.e.*, 1.5 g/kg/day) resulted in a greater anabolic response at whole-body level and MPS. Strikingly, the positive anabolic response achieved with both levels of protein intake was largely due to reductions in protein breakdown, indicating the importance of simultaneous determination of both protein synthesis and breakdown, as protein synthesis actually declined with 0.8 g protein/kg/day, regardless of the distribution patterns. Furthermore, the same study [45] showed that whole body anabolic response increased linearly with increasing amount of protein intake (dose range: ~6.4–91.7 g), without evidence of plateau in older adults [45]. These results extended previous findings shown by the Deutz group [46,47], indicating that the amount of total protein, but not the pattern of protein intake, is of importance with respect to maximizing anabolic response. Importantly, the linear relationship between the amount of protein intake and anabolic response has been recognized for more than half a century, as determined by a nitrogen balance technique, although the anabolic response beyond RDA for protein (*i.e.*, 0.8 g protein/kg/day) has been ignored [48]. Therefore, data indicate that there is no practical limit to the anabolic response in increasing amount of dietary protein intake.

Taken together, the data do not support the notion that a maximal anabolic response is stimulated with ~35 g of high quality protein per meal [23] or 0.4 g/kg/meal (1.2 g/kg/day) for older adults [19]. The “even distribution hypothesis” was based on this limit of anabolic response [40], but that hypothesis ignored many important factors in determining true net anabolic response. These factors include the quality of protein consumed, the contribution of protein breakdown to the net anabolic response, and the potential involvement of whole body response, all of which result in the considerable underestimation of the maximal anabolic response. It is therefore unreasonable to base recommendations for the optimal level of protein intake in elderly adults on the idea that the maximal effective dose of protein is ~35 g per meal. If the goal of the optimal level of protein intake is considered to be the amount needed to maximally stimulate protein anabolism (*i.e.*, synthesis minus breakdown), then consumption of dietary protein in accord with the higher end of the AMDR (35% of total calories) is reasonable. Unfortunately, long-term studies assessing the effect of this level of dietary protein consumption on functional outcomes in elderly adults have not been performed.

### 2.6. Dietary Protein and Anabolic Signaling in Muscle of Elderly Adults

Signaling through mTORC1 is involved in the regulation of several anabolic processes in the body including protein synthesis [26,49,50]. In skeletal muscle, amino acids signal through mTORC1 to initiate the process of protein synthesis [25,51,52,53]. The translation initiation factors 4E-BP1 (eukaryotic initiation factor 4E binding protein 1) and p70S6K (ribosomal protein S6 kinase) are downstream targets of mTORC1 [51,52,53]. Signals provided by EAA, especially leucine, are required for full activation of this pathway [25,51,54]. Muscle becomes resistant to the normal stimulatory effects of postprandial leucine concentrations with increasing age [18], which may result in the reduced stimulation of the mTORC1 pathway and reduced activation of translation initiation and subsequent MPS. This could be due to a reduced sensitivity to leucine with age, to less efficient absorption of leucine from the gut, or to the fact that the dietary protein intake tends to decrease with age [8,55,56].

Age-related muscle loss may involve a decreased response to EAA due to decreased phosphorylation of mTORC1 and p70S6K [22]. In response to 10 g of EAA, mTORC1 phosphorylation, or activation, while significantly increased in skeletal muscle of elderly adults, is still significantly lower in younger adults [22]. Guillet *et al.* [57] found that p70S6K phosphorylation is not stimulated in older adults after infusion with leucine. These findings are supported by Fry *et al.* [58] who found that elderly adults, compared with young adults, have significantly reduced phosphorylation of mTORC1 and translation initiation factors after a bout of resistance exercise. Gene expression of proteins associated with muscle protein synthesis and satellite cell function also differ between young and elderly adults in response to exercise and supplementation with EAA [59]. While no difference was found between young and elderly in the fasted state, there was a significant decrease in protein (REDD1, TSC1, TSC2, and IGF1 receptor) expression six hours post-exercise and EAA intervention in elderly adults *versus* young adults [59]. In addition, after only seven days of bed rest, elderly adults had a reduced response to EAA ingestion resulting in no increase in MPS, activation of translation initiation factors (4E-BP1 and p70S6K), and no increase in amino acid transporters [60]. Elderly adults also had decreased LAT1 (L-type amino acid transporter) and SNAT2 (sodium-coupled neutral amino acid transporter 2) following seven days of bed rest [60]. These findings are further supported in a study by Tanner *et al.* [61], who found that, after five days of bed rest, elderly adults (but not younger adults) had reduced amino acid-induced anabolic sensitivity, resulting in decreased muscle protein synthesis. In this study, elderly adults had increased MURF1 gene expression at baseline and increased AMPKα phosphorylation after bed rest, which is suggestive of increased muscle protein breakdown [61]. These data are important because they demonstrate how quickly an injury or hospital stay could decrease skeletal muscle function. While all of these data suggest a potential role of changes in sensitivity of mTORC1 and related factors in the anabolic response as well as anabolic resistance in elderly adults, it must also be acknowledged that the nature of the data is correlational and thus does not definitively prove a cause–effect relationship. To this end, it has recently been shown that consumption of a very small dose of EAA (3 g) can stimulate muscle protein anabolism equivalently to 20 g of whey protein in the absence of any time-coincident changes in initiation factor activity [62].

## 3. Conclusions

Elderly adults are less responsive to the anabolic stimulus of low doses of amino acid intake compared to younger adults [18]. However, this lack of responsiveness in elderly adults can be overcome with higher levels of protein consumption [18]. This is also reflected in studies comparing varying levels of protein intake [19]. This suggests that the lack of muscle responsiveness to lower doses of protein in older adults can be overcome with a higher level of protein intake. The requirement for a larger dose of protein to generate responses in elderly adults similar to the responses in younger adults provides the support for a beneficial effect of increased protein in elderly populations [8]. The consumption of dietary protein consistent with the upper end of the AMDRs (as much as 30%–35% of total caloric intake) may prove to be beneficial, although practical limitations may make this level of dietary protein intake difficult. The consumption of high-quality proteins that are easily digestible and contain a high proportion of EAAs lessens the urgency of consuming diets with an extremely high protein content.

## Abbreviations

The following abbreviations are used in this manuscript:

4E-BP1	eukaryotic initiation factor 4E-binding protein 1
AMDR	acceptable macronutrient distribution range
AMPK	AMP-activated protein kinase
BMR	basal metabolic rate
DRI	dietary reference intake
EAA	essential amino acids
EAR	estimated average requirement
LAT1	L-type amino acid transporter
MPS	muscle protein synthesis
mTORC1	mechanistic target of rapamycin
MURF1	muscle RING-finger protein-1
p70S6K	ribosomal protein S6 kinase
RDA	recommended dietary allowance
REDD1	regulated in development and DNA damage responses 1
SNAT2	sodium-coupled neutral amino acid transporter 2
TSC1	tuberous sclerosis 1
TSC2	tuberous sclerosis 2
